# The size of larval rearing container modulates the effects of diet amount and larval density on larval development in *Aedes aegypti*

**DOI:** 10.1371/journal.pone.0280736

**Published:** 2023-01-25

**Authors:** Alima Qureshi, Elizabeth Keen, George Brown, Lauren Cator

**Affiliations:** 1 Department of Life Sciences, Imperial College London, Ascot, United Kingdom; 2 Faculty of Science, Agriculture, and Engineering, Newcastle University, Newcastle upon Tyne, United Kingdom; Fundacao Oswaldo Cruz Instituto Rene Rachou, BRAZIL

## Abstract

Mass-rearing of mosquitoes under laboratory conditions is an important part of several new control techniques that rely on the release of males to control mosquito populations. While previous work has investigated the effect of larval density and diet amount on colony productivity, the role of the size of the container in which larval development takes place has been relatively ignored. We investigated the role of container size in shaping life history and how this varied with density and food availability in *Aedes aegypti*, an important disease vector and target of mass-rearing operations. For each treatment combination, immature development time and survival and adult body size and fecundity were measured, and then combined into a measure of productivity. We additionally investigated how larval aggregation behaviour varied with container size. Container size had important effects on life history traits and overall productivity. In particular, increasing container size intensified density and diet effects on immature development time. Productivity was also impacted by container size when larvae were reared at high densities (1.4 larva/ml). In these treatments, the productivity metric of large containers was estimated to be significantly lower than medium or small containers. Regardless of container size, larvae were more likely to be observed at the outer edges of containers, even when this led to extremely high localized densities. We discuss how container size and larval aggregation responses may alter the balance of energy input and output to shape development and productivity.

## Introduction

*Aedes aegypti* is an important vector of the viruses which cause dengue, chikungunya and Zika. These infections are important emerging [1] and re-emerging [2] public health threats. The dengue virus alone threatens over 3.9 billion people in more than 100 countries [3]. Prophylactic and therapeutic options are limited for many of these infections and control of *Ae*. *aegypti* populations is a key part of disease prevention [4]. Until recently, population control has heavily depended on chemical insecticides. These control tools are effective, but are currently threatened by the development of wide-spread insecticide resistance [4]. Alternative strategies involving mass-releases of laboratory reared mosquitoes are rapidly becoming a key tool in the management of *Aedes* populations with releases of mass-reared *Aedes* in the Cayman Islands [5, 6], Brazil [7], Cuba [8], Malaysia [9], China [10] and Singapore [11].

Generally, these strategies involve rearing mosquitoes in the laboratory which carry either a symbiont or a genetic construct designed to reduce the wild population or curtail its ability to transmit disease [12–15]. Production of large numbers of high-quality insects for field release is key for many mass-release strategies [16]. In mosquitoes, the environment experienced in the aquatic larval stage determines the number of adults successfully produced and can have important effects on adult fitness traits [17, 18]. Thus, understanding the regulators of immature development and survival has important implications for colony productivity and the fitness of the adults produced [19].

Diet availability (mg/ml) and larval density (larva/ml) are both important determinants of larval development and adult traits and many studies have focused on these variables in rearing environment [18, 20]. Decreasing larval diet amount has been demonstrated to increase development times [18, 21–28] and decrease larval survival [21, 24, 25] and adult body size [21, 23, 25, 26]. Most studies have reported that higher larval densities result in increased larval development times [23, 29–38] and decreased larval survival [23, 37–40]. Adults emerging from high density conditions tend to be smaller [29–33, 35–37, 39–42] with shorter lifespans [18, 32, 40]. While multiple mechanisms could be responsible for the observed density effects [43–46], work has emphasized the role of competition for nutrient resources associated with increasing density [18, 47].

Recent work indicates that container size may also be an important factor determining immature development and performance. For example, Parker and colleagues investigated the effect of container size on inter and intraspecific competition in *Ae*. *aegypti* and *Ae*. *albopictus*. They found that the effect of intraspecific competition on *Ae*. *aegypti* was compounded in large containers and container size negatively affected larval survival and development rate [48]. Increasing container size could affect larval growth and survival by inhibiting the ability of larvae to locate and access food as well as by increasing energetic demands. In the previous experiment, competition was varied by manipulating food availability measured as mg/larva which resulted in larger containers containing less nutrients per unit volume (mg/ml) than smaller ones [48]. Thus, the mechanisms behind container size effects and their interaction with density (larva/ml) and food availability (mg/ml) remain unclear.

We conducted a study to determine effects of container size, density and diet amount on immature and adult traits in *Ae*. *aegypti*. First, similar to previous work, we varied diet amount available per larva (mg/larva) across container types while keeping larval density (larva/ml) constant. In a second experiment, we varied the density of larvae (larva/ml) across container sizes and provided them with a surplus of diet (mg/larva) at a consistent concentration of diet (mg/ml). We measured larval survival and development along with adult body size and fecundity in these treatments. These life history parameters were then combined to calculate combined measure of productivity for each treatment. To further investigate how larval aggregation behaviours in different conditions may impact development, we photographed larvae to determine how larvae utilized space in different containers at low and high densities. We predicted that the effects of food availability and density would vary with container size to affect larval development and could affect adult traits such as body size and fecundity and ultimately, predicted productivity.

## Materials and methods

### Experimental treatments

*Ae*. *aegypti* (Florida strain, F15-19 originating from Fort Myers, Florida) eggs were hatched under a vacuum for 1500 s, provided with a pinch of ground fish diet (Cichlid gold, Hikari, Kasai, Japan). At 20 hours post-hatch, 1^st^ instar larvae were pooled and counted into small, medium, and large containers (Table 1).

**Table 1:** Three diet treatments were provided to larvae held in three container sizes. Small (10.00 x 4.20 cm; Diameter x Depth), medium (18.00 x 11.00 x 6.00 cm; Length x Width x Depth) and large (27.00 x 20.70 x 11.00 cm; Length x Width x Depth).

Diet	Container size	N	Water Volume (ml)	No. of larvae	mg/larva/day	mg/ml	larva/ml
High	Large	5	1000	200	0.50	0.10	0.20
	Medium	5	500	100	0.50	0.10	0.20
	Small	5	100	20	0.50	0.10	0.20
Medium	Large	5	1000	200	0.30	0.06	0.20
	Medium	5	500	100	0.30	0.06	0.20
	Small	5	100	20	0.30	0.06	0.20
Low	Large	5	1000	200	0.10	0.02	0.20
	Medium	5	500	100	0.10	0.02	0.20
	Small	5	100	20	0.10	0.02	0.20

First, we counted larvae into containers to maintain a constant density of 0.20 larva/ml. We manipulated diet amount available in these containers by providing larvae with 0.10, 0.30, 0.50 mg diet/larva daily (hereafter referred to as low, medium, and high) each day until the last larvae pupated. Diet was powdered finely to avoid particles settling on the floor of the container and was distributed evenly across the container. There were five replicate containers per food amount (Table 1).

Secondly, larvae were reared using these same container sizes at either low (0.20 larvae/ml) or high (1.40 larvae/ml) density. These densities are in line with previous studies capturing density dependent effects in *Ae*. *aegypti* (S1 Table). In pilot experiments, there was no increase in winglength when larvae were provided more than 0.70 mg/larva/day (S1 Fig). Trays were provided with a surplus of diet (>3.5 mg/larva/day). This is seven times the amount of diet/ml presented in the ‘high’ diet treatment from the first experiment. After 7 days, water from containers was replaced each day prior to feeding to prevent fouling [21]. There were four to five replicates per density/container size treatment (Table 2). In all experiments, the diet in each tub was spread evenly across the surface of the water.

**Table 2:** Summary of treatment conditions for density experiments. Small (10.00 x 4.20 cm; Diameter x Depth), medium (18.00 x 11.00 x 6.00 cm; Length x Width x Depth) and large (27.00 x 20.70 x 11.00 cm; Length x Width x Depth).

Treatment	Container Size	N	Water Volume (ml)	No. of larvae	mg/larva/day	mg/ml	larva/ ml
High	Large	4	1000	1400	5.00	7.00	1.40
	Medium	4	500	700	5.00	7.00	1.40
	Small	5	100	140	5.00	7.00	1.40
Low	Large	4	1000	200	3.50	7.00	0.20
	Medium	4	500	100	3.50	7.00	0.20
	Small	5	100	20	3.50	7.00	0.20

### Immature and adult life history traits

Pupation, eclosion and survival were recorded by counting pupae and emerged adults daily. Pupae were transferred to 19.00 cm x 19 cm x 19 cm plexi-glass holding cages for emergence. Adults emerging from each tray were counted, sexed, aspirated into separate holding cages for mating, and provided with 10% sucrose solution daily. All individuals were left to mate in treatment and replicate-specific holding cages for 7 days post eclosion. Sucrose was removed at 36 hours prior to blood feeding, to starve females. At 36 hours, all replicates were provided defibrinated horse blood (First Link (UK) Ltd.) via a membrane feeding system (Hemotek Ltd.), using natural hog sausage casing as the membrane (Weschenfelder Direct Ltd.) (Approved by Health and Safety, Imperial College London). Four to six blood fed females from each replicate were randomly selected and placed in individual, 50 ml Falcon fecundity assay tubes modified for ovipositon. The lids were drilled out and a damp filter paper folded into a cone fitted to the bottom of the tube. Tubes were then placed on custom-built racks to allow them to be partially submerged in water. After the bloodmeal, fecundity assays were left to run for 10 days, in order to enable all eggs to be laid from the first gonotrophic cycle. The females used in the fecundity assays were frozen overnight and their right wing was removed and right winglength was used as a proxy for body size. Previously, studies have shown a strong correlation between winglength and body size [49, 50]. After removal, the right wing was measured from the distal end of the alula to the end of the longest part of the wing, excluding the fringe setae.

### Productivity

We incorporated our trait measures into a single measure of productivity based on previous studies [51–53].

$$\text{Productivity}=\frac{\ln(Fx100)xS)}{D}$$

*F* represents mean fecundity of each population (we assumed 100% hatch rate), *S* represents the mean proportion of larvae surviving to adulthood and *D* is representing the mean larval development time in days.

### Image analysis of larval distribution in density treatments

**Fig 1:**
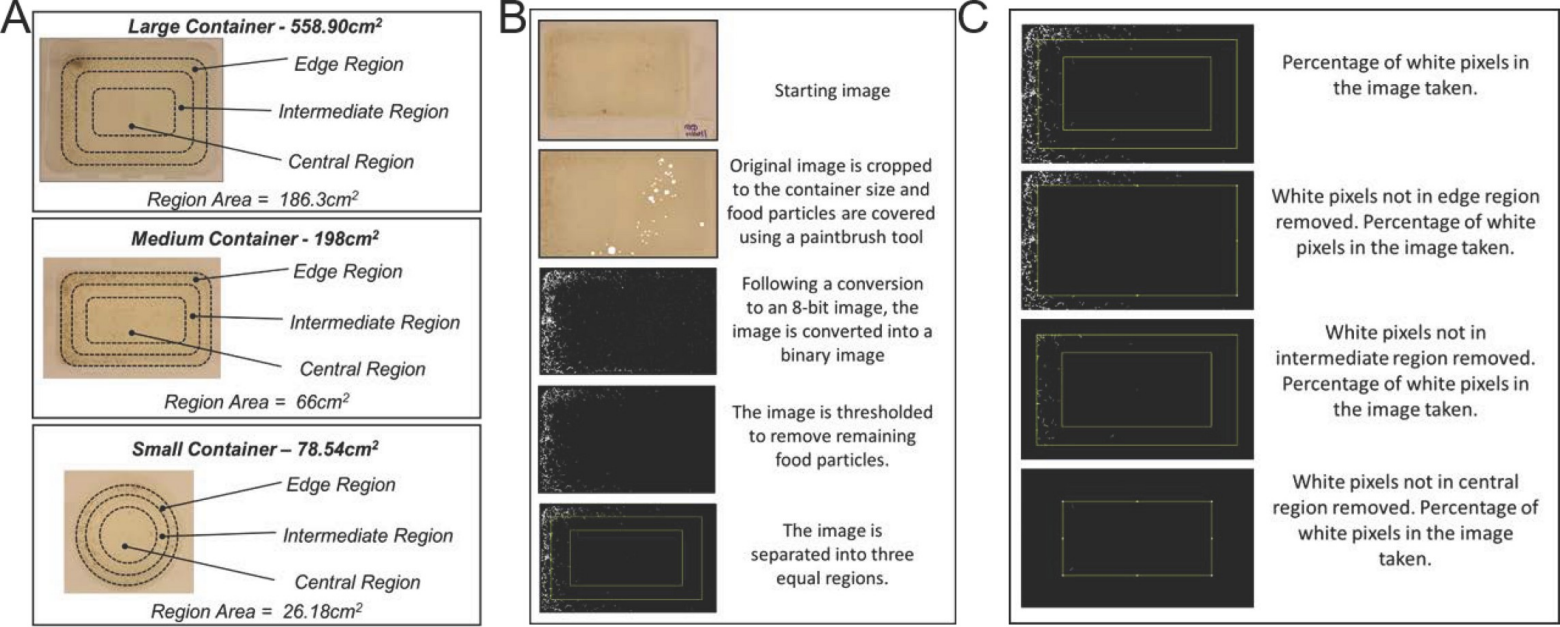
A. The relative areas of the three regions for each of the differing container sizes. Container sizes are labelled with the relative area size for their three regions. Large containers had regions measuring 186.30 cm^2^, for medium containers 66.00 cm^2^ and for small containers 26.18 cm^2^ B. Processing of images to allow counts for each region. C. Example of calculation of percentage of larvae in outer, intermediate, and central region.

To determine how larvae utilized space in containers under different density conditions, we photographed larvae in four separate sessions, twice during the morning and twice during the afternoon, on days 2–4 of development. In each session, the initial five minutes were used as a settling period, then three containers per treatment were randomly selected and photographed three times, one minute apart. All images were loaded into ImageJ [54] and divided into three equal regions (Fig 1A). Each container included; an edge region, which touched the wall of the container, central region, which included the area of water in the centre of the container, and an intermediate region was selected which included the water between the edge and central regions (Fig 1A).

For processing, images were cropped to the area of the water surface and food particles were manually coloured with a white paintbrush tool. Next, the image was inverted, to highlight the larvae as bright against the dark background. The image was converted into an 8-bit format, making it possible to threshold the image. For each image the threshold was set so as to remove background noise (small food particles and waste) but maintain detection of larvae. The ‘despeckle’ and the ‘pencil’ tools [54] were used to remove remaining food particles from the analysis. Finally, the image was converted to binary for area fraction analysis of white and black. The regions were overlaid onto the processed image and fraction of the area occupied by larvae was calculated for each region and the total occupied area of the container (Fig 1).

First, we calculated the larval coverage per region as larval pixels divided by total pixels in each region. The manual cropping of images and overlaying of regions resulted in each region differing slightly in total pixel number. We corrected for these errors by multiplying the larval coverage for each region by the total pixels of the region divided by the total pixels of the image. This provided the corrected larval coverage for each region, which we then divided by the sum of all three corrected larval regional coverage (total larval coverage). This produced the larval regional preference for each region as a fraction. These fractions were then multiplied by the total number of larvae per treatment and divided by a third of the water volume of the container to calculate the density of larvae in each region.

### Statistical analyses

All analysis was conducted in R version 3.5.1 (R Core Team 2018). To assess the effect of treatments (density, diet and container size) on development we used a Generalized Linear Mixed Effects Model (GLMM) fit with a Poisson distribution (package ‘afex’, [55]). The response variable was the day of pupation with container size and either diet or density treatment along with their interaction included as fixed effects and replicate included as a random effect. The effect of treatments on survival was assessed using a GLMM with a Gamma family log link function. The response variable was the percentage of individuals surviving to adulthood with experimental treatments as fixed effects (container size, diet or density and their interaction). A Linear Mixed Effect Model (LMM) with replicate as a random effect was used to establish whether there was a difference in female winglength between the treatments (package ‘lme4’; [56]). A negative binomial GLMM with replicate as a random effect was used to account for over-dispersion to determine if there was a difference in the number of eggs laid between treatments. Container and diet or density treatment were incorporated as fixed effect with wing length incorporated as a covariate. The effect of diet on productivity was determined using an LMM with the cubed productivity value as the response variable, diet and container size treatment as fixed effects, and replicate as a random factor. The effect of density on productivity was determined using an LMM with the sin transformed productivity value as the response variable, density and container size treatments as fixed effects, and replicate as a random factors. In all cases, Tukey post hoc comparisons were used to determine pairwise differences between the treatments (package ‘Ismeans’, [57]).

We investigated the sensitivity of productivity to *D*, *F*, and *S*. Simulated productivity values were calculated by holding either *D*, *S*, *F* or *SF* variables constant. For each simulated productivity, we ran a LMM to determine the effect of container size and diet or density and their interaction. We also calculated an effect size (η^2^) for each variable by dividing the sum of squares associated with the variable of interest by the total sum of squares [58].

A repeatability analysis [59] was carried out to see if images taken of the same containers within 1 minute (photoshoot) of each other produced similar results. We determined the repeatability of larval preferences for the edge, intermediate and central regions across replicates by fitting a mixed model (GLMM) using Markov chain Monte Carlo techniques using replicate as a random effect and 5,000,000 iterations. We determined if the proportion of larvae observed in the central, intermediate, and edge region differed significantly within a density treatment we used Kruskal-Wallis test with a post-hoc Dunn test for pairwise comparisons [60]. We determined the effect of density, and container size on the proportion of larvae in each region using separate linear mixed-models LMM [56]. Both density and container size were used as interacting fixed effects, with replicate as a random effect. A type-III ANOVA with Satterthwaite’s method [57] was applied to the models to see if treatment produced significant differences in the larval preference of the three differing regions. The same separate models were fitted with the regional densities (larvae/ml) calculated as response variables (LMM). Both density and container size were used as interacting fixed effects. Following this, a type-III ANOVA was used. All raw data are included in S1–S11 Datasets.

## Results

### Manipulation of container size and diet

**Fig 2:**
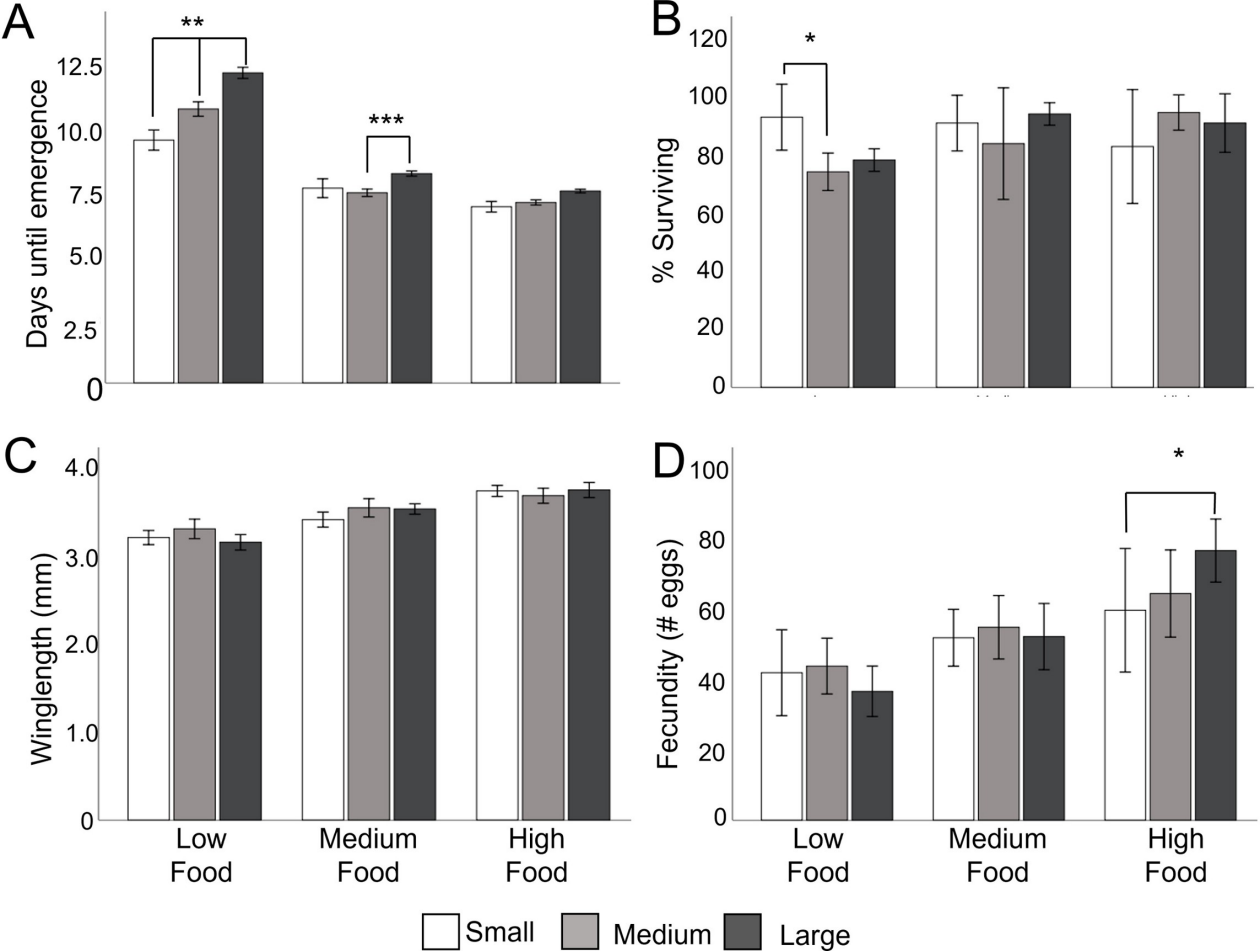
The effect of diet and container size on development, size, and fecundity. **A. Days from hatching to emergence of adult mosquito**. Numbers averaged across the five replicate containers for each container size for each diet type. There were a total of n = 20, n = 100 and n = 200 individuals, for each treatment/replicate, for small, medium and large containers respectively. **B. Percentage of first instar larvae surviving to adult emergence** for 5 replicate trays per treatment. **C. Female winglength** across replicate trays for each container size (number of females per treatment; high diet; large = 20, medium = 20, small = 24, medium diet; large = 20, medium = 19, small = 27, low diet; large = 20, medium = 20, small = 29). **D. Eggs laid per female**. Number of eggs laid by each female averaged across the five replicates for each container size for each diet type (number of females/treatment; high diet; large = 17, medium = 16, small = 11, medium diet; large = 18, medium = 18, small = 10, low diet; large = 16, medium = 20, small = 8). Error bars represent ± SE. * = P<0.05, ** = P<0.01 and *** = P<0.0001 from Tukey post hoc pairwise comparisons between container size treatments within the same diet treatment.

Across all container sizes increasing diet reduced larval development time (GZLM, Diet, ֛χ^2^ = 122.41, df = 2, P<0.001; Fig 2A) and increased the size of emerging females (֛F = 71.76, df_num_ = 2, df_den_ = 38.75, P<0.0001; Fig 2C). Larger females produced more eggs (χ^2^ = 41.99, df = 1, P<0.001), but diet did not directly affect fecundity when winglength was controlled for (֛χ^2^ = 5.34, df = 2, P = 0.07; Fig 2D). We did not find a significant effect of diet on immature survival (χ^2^ = 3.95, df = 2, P = 0.14; Fig 2B).

Container size modulated the strength of the diet effect on larval development time (GZLM, Diet x Container, χ^2^ = 14.26, df = 4, P<0.001). When larvae were fed on the low diet, increasing container size increased the average time it took for larvae to develop. Larval development increased on average 1.2 and 2.7 days in large containers compared to medium or small containers respectively (Fig 2A). As diet amount increased, the effect of container size weakened, but was still present (Fig 2A). There was no significant effect of container size or interaction with container size and diet on survival (container size, χ^2^ = 1.28, df = 2. P = 0.53; diet x container size, χ^2^ = 8.34, df = 4, P = 0.08), fecundity (container size, χ^2^ = 0.99, df = 2, P = 0.61; diet x container size, χ^2^ = 6.05, df = 4, P = 0.20) or emerging female size (container size, F = 0.91, df_num_ = 2, df_den_ = 38.44, P = 0.41; diet x container size, F = 0.91, df_num_ = 2, df_den_ = 38.41, P = 0.41).

**Fig 3:**
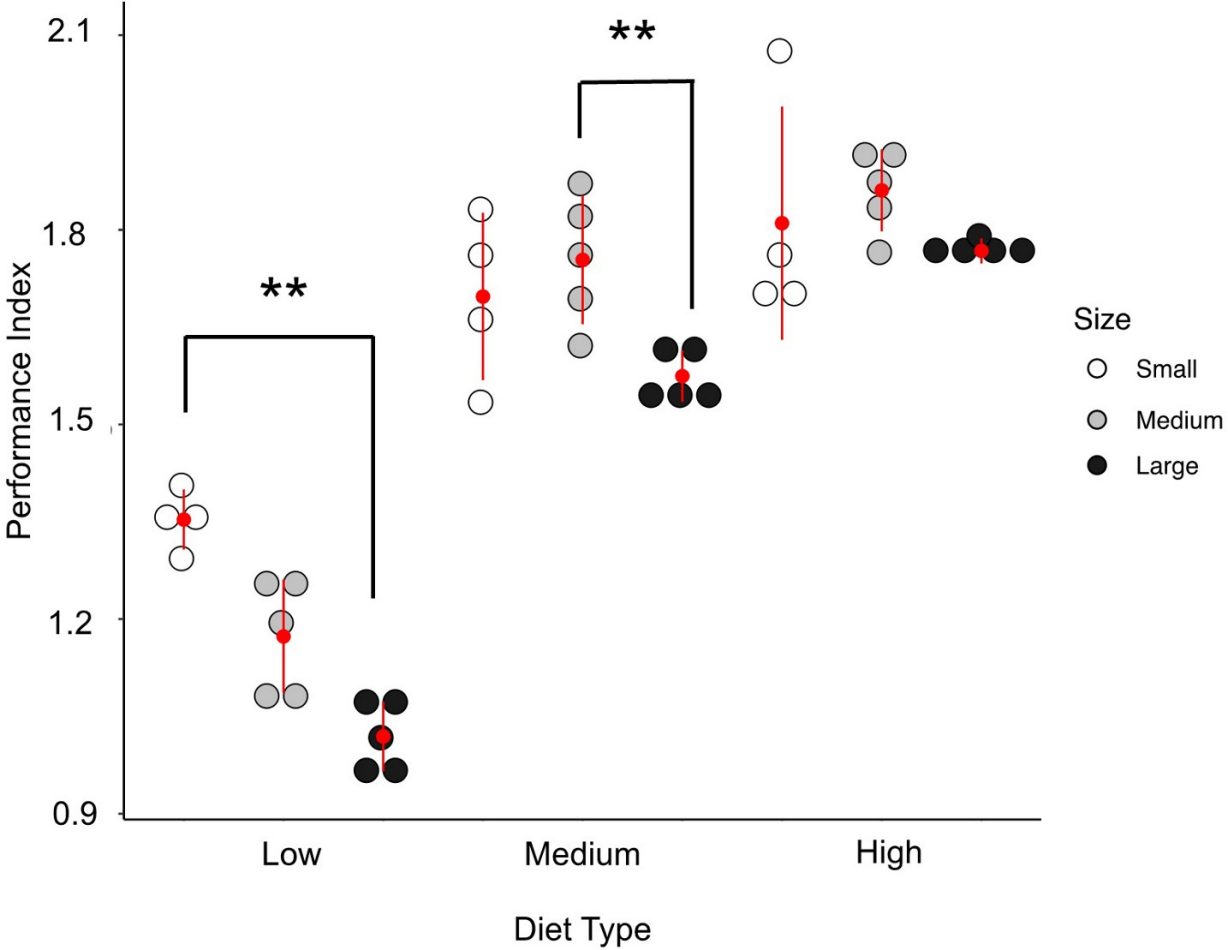
Productivity for each container size and diet type. The individual points represent the productivity for each replicate with the red dot representing the mean productivity for that treatment (n = 5 trays/treatment). The red line either side of the mean represents ± 1 SD.

When life history traits from each replicate tray were used to calculate productivity, we found that across all container sizes, increasing diet resulted in an increase in productivity (F = 131.52, df_num_ = 2, df_den_ = 29.19, P<0.001, η^2^ = 0.91). There was also a much smaller but significant effect of container size on the productivity (F = 9.74, df_num_ = 2 df_den_ = 29.52, P<0.001, η^2^ = 0.068) with large containers exhibiting a lower productivity than small or medium containers (Tukey, posthoc comparisons, P<0.01) (Fig 3). There was not a significant interaction between container size and diet amount per ml (F = 1.45, df_num_ = 4, df_den_ = 29.19, P = 0.24, η^2^ = 0.02). The effect of diet was robust to variation of all variables, while the effect of size on productivity was sensitive to variation in *D* (S2 Table). When *D* was held constant the small but significant effect of container size on productivity was eliminated.

### Manipulation of container size and density

**Fig 4:**
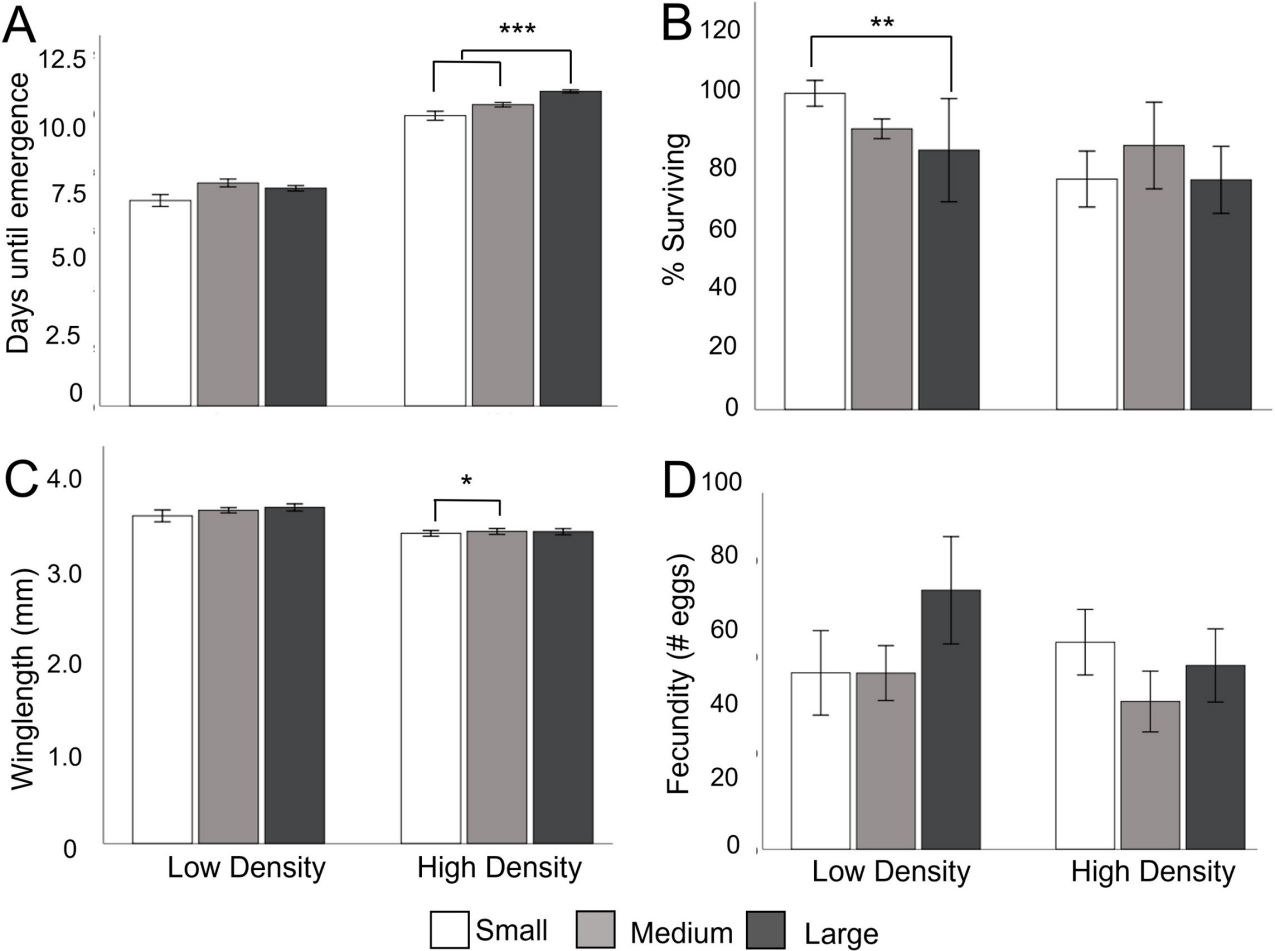
The effect of density and container size on development, size, and fecundity. A. Days from hatching to emergence of adult mosquito. Numbers averaged across the five replicates for each container size for each diet type. There were a total of n = 20, n = 100, and n = 200 individuals, for each replicate, for small, medium and large containers respectively for low density conditions and n = 1400, n = 700, and n = 140 for each replicate for small, medium, and large containers for high density conditions. **B. Percentage of first instar larvae surviving to adult emergence**. Proportions taken from 4 replicate trays for large and medium contains and 5 for small containers. **C. Female wing length** across replicate trays for each container size (sample size were high density; small = 33, medium = 28, large = 28, low density; small = 25, medium = 18, large = 25). **D. Eggs laid per female.** Numbers averaged across the five replicates for each container size for each diet type. (sample sizes were high density; small = 32, medium = 27, and large = 27. low density; small = 21, medium = 16, large = 22). Error bars represent ± SE. The bars and asterisks represent significant pairwise differences from a Tukey’s post doc test where * = P<0.05, ** = P<0.01 and *** = P<0.0001 between container size treatments within the same density treatment.

Larvae reared in high density treatments emerged later that those in low density treatments (χ^2^ = 72.73, df = 2, P<0.0001). Development time also increased with container size (χ^2^ = 10.32, df = 2, P = 0.006). There was an interaction between container size and density such that the effect of container size increased at higher densities (χ^2^ = 6.02, df = 2, P<0.05; Fig 4A). Immature survival was significantly lower in the high density containers (χ^2^ = 12.71, df = 1, P<0.001; Fig 4B) and did not change with container size (χ^2^ = 5.42, df = 2, P = 0.07). Females emerging from high density containers were smaller (χ^2^ = 32.77. df = 1, P<0.001; Fig 4C) and female size did not vary significantly with container size (χ^2^ = 3.30, df = 2, P = 0.19). Again, while larger females produced more eggs (χ^2^ = 4.02, df = 1, P = .05), neither density (χ^2^ = 0.02, df = 1, P = 0.90) or container size (χ^2^ = 3.82, df = 2, P = 0.15) directly affected fecundity when wing length was incorporated as a covariate (Fig 4D).

**Fig 5:**
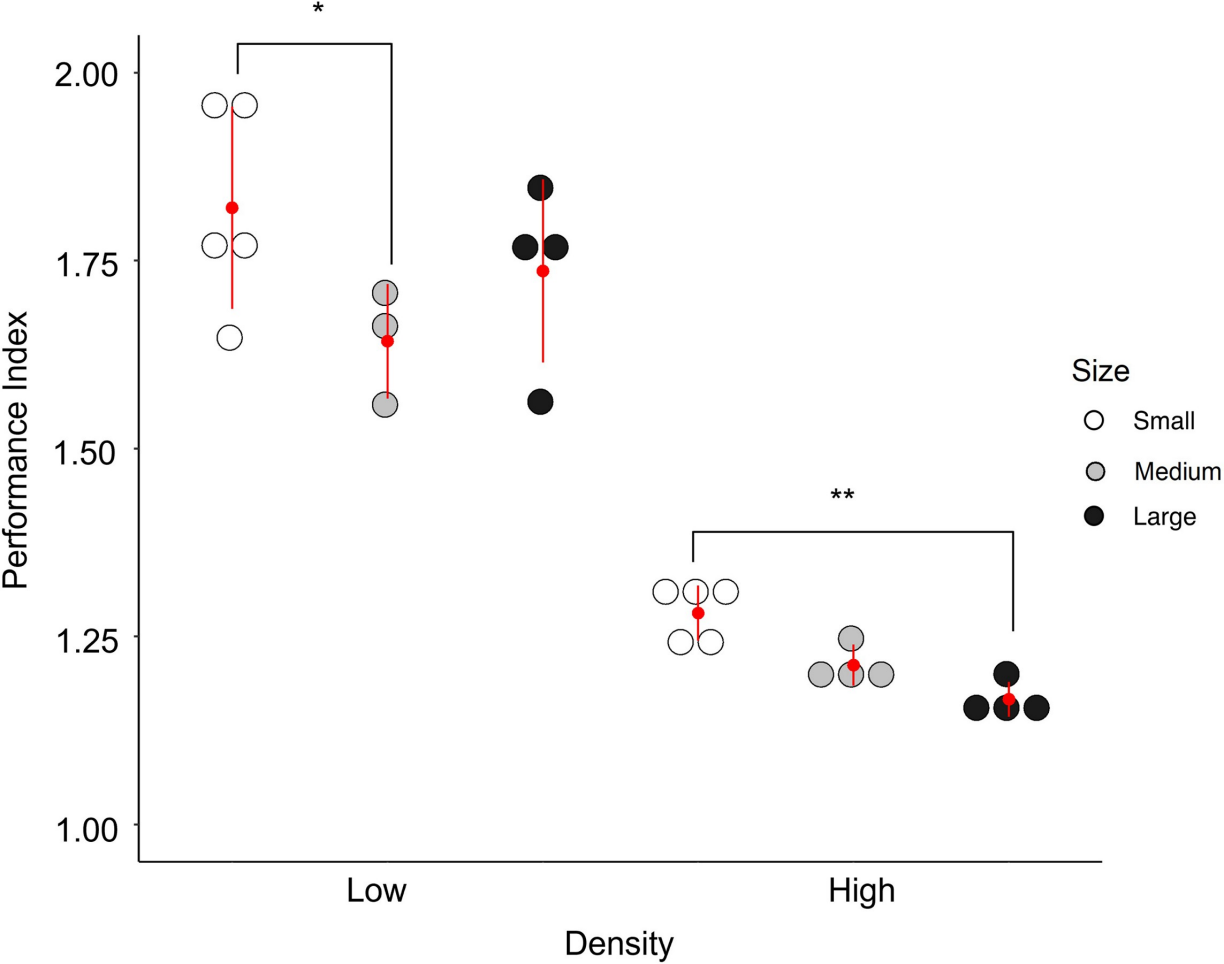
Productivity for each container size and density. The individual points represent the productivity for each replicate with the red dot representing the mean productivity for that treatment (n = 4–5 trays/treatment). The red line either side of the mean represents ± 1 SD.

High density containers across treatments had lower productivity values (F = 36.67, df_num_ = 1, df_den_ = 15.62, P<0.001, η^2^ = 0.65) and container size interacted with density (F = 8.26, df_num_ = 2, df_den_ = 05.61, P<0.01, η^2^ = 0.29; Fig 5) such that at low densities there were no differences in container size performance, but in high density treatments large containers were significantly less productive than small containers (Tukey post-hoc comparison, P = 0.01). The same results were obtained when *F* and *S* were held constant and *D* varied as we observed in the experiment. When *D* was held constant both the main effect of density and the interaction between density and container size decreased in how much variance they explained and lacked statistical significance (S2 Table).

### Larval distribution in density treatments

The percentages of larvae in each region were highly repeatable across all regions within images that made up replicates (central, *R* = 0.79, CI = 0.71–0.86; intermediate, *R* = 0.82, CI = 0.76–0.88; edge, *R* = 0.88, CI = 0.82–0.92). Across all treatments, our analysis indicates that larvae were found more frequently in the edge region of the container with 75.94% ± 0.22 (mean ± SE) of larvae on average observed in this region (Kruskal-Wallace Test; χ^2^ = 468.06, *P*<0.0001). Pairwise comparisons with the Dunn-test indicated that significantly more larvae were observed in the edge region of the container compared to the central (*P*<0.0001) and intermediate region (*P*<0.0001).

Both intermediate and central regions also differed significantly in the percentage of larvae observed there with 15.05% ± 0.17 in the intermediate and 9.00% ± 0.18 in the central region of the container (*P*<0.0001). There was no significant effect of either density (Type III Anova; F_1,19.9_ = 1.91, *P* = 0.18) or container size (F_1,19.9_ = 0.28, *P* = 0.76) on the larvae’s preference for the edge.

As expected, larval density was a significant predictor of regional density in the edge (F_1,19.8_ = 1146.62, *P* <0.0001), intermediate (F_1,19.5_ = 178.82, *P*<0.0001), and central (F_1,24.8_ = 18.80, *P*<0.001) regions. There was no effect of container size or interaction between container size and density treatment. The localized density of larvae in the edge region of containers was more than double of the density expected if they were evenly distributed, while the localized density in the intermediate and central regions was much lower (Table 3).

**Table 3:** Mean density of larvae observed in each region of containers (± 1 SE).

	High Density Treatment	Low Density Treatment
	(larvae/ml)	(larvae/ml)
	n = 105	n = 108
**Edge Region**	3.25 ± 0.06	0.45 ± 0.02
**Intermediate Region**	0.64 ± 0.05	0.08 ± 0.02
**Central Region**	0.31 ± 0.05	0.63 ± 0.02

## Discussion

We measured the effect of container size on larval development and survival. We found that container size affected larval development and estimated productivity both when diet availability and larval density were varied. In high density and low diet availability treatments, larvae took ~1–3 days longer to emerge when held in large compared to small containers. We next combined effects on different aspects of life history into a productivity metric. When we varied diet amount available (mg/ml), we found container size had a small but significant effect on overall performance independent of how much diet was available. In containers where density was varied (larvae/ml), container size interacted with density and had a large and significant compounding effect on productivity. The interaction between density and container size was observed despite the fact that diet was in surplus and at a consistent concentration in the trays, suggesting that intensified competition for food among larvae isn’t the only mechanism underlying the observed effect of container size. Standard mass-rearing practices suggest higher estimated larval densities and higher amounts of diet per larva, but not per ml of water [61]. Thus, we would expect that similar sort of container size effects occur in these conditions. Critically, the interactions between density and container size were observed despite very high levels of food availability. This suggests that the effects of container size at high densities in mass-rearing operations cannot be mitigated by adding more diet to these containers. Combined with our observations about how larvae distribute themselves in containers, our results suggest that the absolute amount of space available to larvae is a potentially unexplored avenue for optimizing mass rearing operations.

While there has been work indicating that larvae can directly interfere with conspecific growth by producing a growth retardant factor [62], this is unlikely to be the mode of action in our experiment, as this chemical interference primarily occurs when late instar larvae interfere with earlier instars. In this experiment, all larvae were at similar stages of development within containers [62]. We propose that effects of container size on the balance of energetic inputs and outputs are a more likely explanation for the patterns observed here.

One way that container size might affect larval development is by making it more difficult for them to locate and consume food. *Ae*. *aegypti* larvae are opportunistic filter feeders filtering food particles from the water. Recent work suggests that larvae to do not exhibit directed navigation in response nutrient gradients and instead alter their speed when they hit higher pockets of nutrients [63]. There was a small, but independent effect of container size on productivity in diet experiments in which food was available in concentrations of 0.02–0.1 mg/ml. Despite the fact that the powdered food was distributed evenly in our experiments, diet may have been more difficult to locate under food limited conditions as container size increased. In these experiments the low diet concentrations may have also led to greater energetic expenditure. Tracking of individual larvae had revealed that starved animals spend more time searching for food [46].

The most pronounced effects of container size were observed in high density environments. Some of this might be explained by competition for food. Despite the fact that food was in surplus across the container, it may have become locally limiting in the region of the container where larvae were concentrated [23]. Increasing container size may have also increased energy expenditures independent of density. *Ae*. *aegypti* larvae have been observed to dive to find necessary nutrients [64, 65] and repeated diving behaviour required for foraging in food limited environments has been shown to severely reduce individual calorific content [66]. Differences in container and therefore water depth, may also therefore contributed to our results. Few studies have explicitly considered container depth (S1 Table) and future work could address the relative effects of depth and surface area.

We suggest that larval behaviour also played a key role in the observed container effects. *Ae*. *aegypti* have been observed to aggregate along container walls [67–69]. We quantified this behaviour and found that larvae are more likely to utilize the outer edges of containers. While the degree of this aggregation behaviour did not vary with container size, it greatly increased the density experienced by larvae in different regions of containers. Our estimations based on proportion of pixels occupied by larvae are conservative and when larvae overlapped or greatly differed in size may be underestimation of realized density. Previous work has documented that physical contact such as collisions with other larvae may impair larvae from collecting and processing food [62]. At a higher density, the probability of contact was increased, and therefore may have further reduced the ability of larvae to collect food and increased energetic demands associated with both disturbances. Previous work has suggested that these “massing” effects may lead to improper nourishment [21, 67]. Therefore, under high density conditions in large containers local diet limitation, increased energetic demands of foraging, and massing effects may have combined to cause disproportionate effects on development.

The reasons why larvae exhibit such “edge biased” distributions are unknown. Other insects as diverse as almond months [70] and flour beetles [71] have shown these types of spatial distributions in laboratory experiments. Research in the field of spatial ecology has produced several theoretical models which produce these types of patterns depending on different underlying processes [72]. Our data most closely resemble an attractive edge model [73] which assumes the ability of an edge to pull or trap insects. While in field systems heterogeneity in resources, microclimate, or predation risk can pull insects to edges [72], we suspect that in our case, larvae become trapped by the edge either due to limit on locomotion or navigation. This explains why they remain at the edge despite the resulting crowding. Further work should be undertaken to understand the sensory responses that lead to these edge biased distributions in captive rearing conditions.

Interestingly, container size has also been shown to alter functional responses to resources in other arthropods [74, 75]. In these cases, container size effects were also driven by how these species and their prey utilize space within containers [74–76]. Our results here, highlight the importance of considering the total space available and the behavioural aspects of how animals utilize that space. This implies that density and diet affect may not simply “scale up” as container sizes are increased and that container size should be controlled for when comparing across experiments. Further, *Ae*. *aegypti* utilizes manmade containers as larval habitats in nature which can vary widely in size [77–79], future work on the edge-biased distributions could be informative for understanding dynamics and heterogeneity in these natural habitats as well.

The productivity metric we chose to use in this study was particularly sensitive to development time, which appears to drive the differences between treatments. For both experiments, simulated productivity values in which both *S* and *F* were held constant so that only *D* varied as we observed experimentally produced effect of similar size and significance (S2 Table). This productivity is a simplification of an original metric developed to approximate population growth (*r*) [51]. In other performance metrics, fecundity contributes more to performance [80]. Further comparison of metrics designed to capture colony productivity would be useful comparing conditions across studies. More broadly, we suggest that metrics used to compare rearing procedures should consider both how many mosquitoes are produced and the likelihood that the males produced by that colony will successfully compete with wild males for mates in the field. Males and females may be differentially affected by colony rearing practices [81, 82]. Ideally, these rearing protocols need to maximize both the number of males produced and their mating effectiveness. As we continue to fill gaps in our understanding of male mosquito biology and which life history traits contribute to male mating success [83], appropriate metrics could be developed to capture and combine both colony productivity and male quality.

Future work on spatial ecology and behaviour, could be used to design rearing vessels that adopt new shapes with varying edge ratios, additional features such as ridges, or other cues that could encourage larvae to distribute themselves more evenly relative to conspecifics and diet. Beyond designing protocols for captive rearing, larval behaviour and development rate have a cemented role in adult population levels and viral development in *Aedes* [84]. Evaluating larval behaviour under a variety of space, diet and density conditions may allow us to determine how larvae respond to changes in microhabitats and competitive environments and the implications of these for population and transmission dynamics.

## Supporting information

S1 Table A summary of some of the current laboratory studies with *A*. *aegypti* demonstrating the variation in the laboratory larvae rearing tray size used. (DOCX)

S2 Table Outputs (degrees of freedom, F statistics, P-values, and effect sizes for all simulated productivity datasets. (DOCX)

S1 Fig Pilot data on the effect of mg/larva/day on adult male and female body size. (DOCX)

S1 Dataset The effect of density of development time. ID = individual mosquito ID, Container = Container Size (Large/Medium/Small), Density = Density (Low/High), Number = Replicate Tray, Day = Day post hatch that mosquito emerged as adult.
(CSV)

S2 Dataset The effect of density on survival. Size = Container Size (Large/Medium/Small), Density = Density (Low/High), Replicate = Replicate (1–4, 1–5 depending on treatment), Tray = individual tray ID, LA = larval to adult survival (%).
(CSV)

S3 Dataset The effect of density on female fecundity and winglength. Size = Container Size (Large/Medium/Small), Density = Density (Low/High), Replicate = Tray ID, MM = winglength of female in mm, Fecundity = number of eggs in first clutch.
(CSV)

S4 DatasetThe effect of density on productivity.Size = Container Size (Large/Medium/Small), Density = Density (Low/High), Replicate = Replicate 1–4, 1–5 depending on treatment, Fecundity = mean fecundity for this replicate tray, Hatch = percentage hatching (assumed to be 100% in this experiment), Survival = proportion of L1 surviving to emerge as adults, Development = mean development time in days, PI = Productivity calculated as $\frac{\ln((100\;x\;F)xS)}{D}$.(CSV)

S5 Dataset The effect of diet on development time. ID = individual mosquito ID, Size = Container Size (Large/Medium/Small), Diet = Amount of Diet (Low/Medium/High), Tray = Tray ID, Replicate = Replicate (1–4, 1–5 depending on treatment) Day = Day post hatch that mosquito emerged as adult.
(CSV)

S6 Dataset The effect of diet on survival. Size = Container Size (Large/Medium/Small), Diet = Diet (Low/Medium/High), Replicate = Replicate 1–4, 1–5 depending on treatment, Tray = individual tray ID, LA = larval to adult survival (%).
(CSV)

S7 Dataset The effect of diet on female fecundity and winglength. Size = Container Size (Large/Medium/Small), Diet = Amount of diet (Low/Medium/High), Number = Female ID used in experiment, Replicate = Replicate (1–4,1–5 depending on treatment), MM_Size = winglength of female in mm, Fecundity = number of eggs in first clutch.
(CSV)

S8 DatasetThe effect of diet on productivity.Size = Container Size (Large/Medium/Small), Diet = Amount of Diet (Low/Medium/High), Tray = Tray ID, Replicate = Replicate 1–4, 1–5 depending on treatment, Fecundity = mean fecundity for this replicate tray, Hatch = percentage hatching (assumed to be 100% in this experiment), Survival = proportion of L1 surviving to emerge as adults, Development = mean development time in days, PI = Productivity calculated as $\frac{\ln(F\;x\;S)}{D}$.(CSV)

S9 Dataset Larval behaviour regional preferences. ID = individual identifier, Replicate = replicate tray, Size = Container Size (Large/Medium/Small), Density = Density (Low/High), Treatment = Combined container size and density treatment, Interval = day and time photo taken (1–4), region- portion of container (edge/intermediate/central), larper = number of larvae in region, larml = number of larvae per ml volume.
(CSV)

S10 Dataset Larval behaviour daily average regional preferences. Three images taken on each day for each replicate were averaged. ID = Image ID, Replicate = Replicate Tray Photographed, Size = Container Size (Large/Medium/Small), Density = Density (Low/High), Treat = Combined Container Size and Density Treatment, Interval = Refers to the day and time in which the photograph was taken (Interval 1 was on the afternoon of the first day, 2 on the morning of the 2^nd^ day, 3 on the afternoon of the 3^rd^ day and 4 on the morning of the 3^rd^ day). Elarper = percentage of larvae in the container than are found in the edge, Mlarper = percentage in the intermediate region, Clarper = percentage found in the central region. Elarml refers to the density of larvae in the edge region (larvae per ml volume of water). Mlarml refers to the density of larvae in the intermediate region (larvae per ml volume of water). Clarml refers to the density of larvae in the central region (larvae per ml volume of water).
(CSV)

S11 Dataset Larval behaviour repeatability. Size = Container Size (Large/Medium/Small), Density = Density (Low/High), Replicate- specific container, replicate1 =, ID =, ID2 =, Date = Date photo taken, edge = area fraction, percentage of large coverage in edge region, central = area fraction, percentage of large coverage in central region, central = area fraction, percentage of large coverage in central region, total coverage = percentage of the entire container was covered in larvae compared to water, eper = percentage of larvae in edge region, mper = percentage of larvae in intermediate region, cper = percentage of larvae in central region, eml = density of larvae in edge region expressed as larvae per ml, mml = density of larvae in intermediate region expressed as larvae per ml, cml = density of larvae in central region expressed as larvae per ml.
(CSV)
