# Supplementary material for: The size of larval rearing container modulates the effects of diet amount and larval density on larval development in Aedes aegypti
Source: PLoS One. 2023 Jan 25;18(1):e0280736. doi: 10.1371/journal.pone.0280736 (PMC9876358; doi:10.1371/journal.pone.0280736)
Supplement: S1 Table — (DOCX) [file pone.0280736.s001.docx]

Supplementary Information:

**Table 2.** A summary of some of the current laboratory studies with *A. aegypti* demonstrating the variation in the laboratory larvae rearing tray size used.

| **Larval rearing tray size** | **Volume of water used per tray** | **Number of larvae per tray** | **Density (Larvae per millilitre)** | **Focus of experiment** | **Traits looked at** | **Reference** |
| --- | --- | --- | --- | --- | --- | --- |
| Unspecified | 250ml | 25 (L3 – no mention of L1 – L2 rearing) | 0.1 larvae/ml | Interactions between high temperature and *Bacillus thuringiensis* subsp. *Israelensis* (Bti) [non-chemical control] | Total protein level and enzyme activity (e.g. Cytochhrome P450 monooxygenas, α-esterase, β-esterase, and Glutathione S-transferase). | Achari et al. (2022) |
| Vial of diameter 20mm x 95mm | 5ml | 1, 2 or 3 | 0.2 larvae/ml, 0.4 larvae/ml, 0.6 larvae/ml | Density | Larvae to adult survival, age at pupation, adult wing length and adult longevity | Agnew et al. (2002) |
| 5 litre container, dimensions unspecified | 4,000ml tap water and 500ml oak leaf infusion water | 320 | 0.08 larvae/ml | The effect of size on susceptibility to infection | Wing size | Alto et al. (2008) |
| Unspecified | 200ml | 40 | 0.2 larvae/ml | Diet | Development time (first instar to adult emergence), larvae to pupae survival, pupae to adult survival, male and female mass and pupae resistance to starvation (number of days alive without food before death) | Arrivillaga and Barrera (2004) |
| Unspecified. However, water surface area given (79cm) with a water depth of 1.5cm | 100ml | 100, 400 and  800 | 1 larvae/ml,  4 larvae/ml,  8 larvae/ml | Density and comparing a genetically modified lines to a  non-modified | Larval mortality, development rate (time to pupation), adult wing size and adult longevity | Bargielowski et al. (2011) |
| **Larval rearing tray size** | **Volume of water used per tray** | **Number of larvae per tray** | **Density (Larvae per millilitre)** | **Focus of experiment** | **Traits looked at** | **Reference** |
| Vial (diameter of 25mm x 95mm) | 5ml | 1 | 0.2 larvae/ml | Diet and virulence across a diet gradient | Time until pupation, larvae to pupae survival, sex, adult longevity, wing length and spore load | Bedhomme et al. (2004) |
| 38 x 25 x 6cm | 500ml | 750 | 1.5 larvae/ml | Diet | Time to pupation, time to adult emergence, sex ratio, adult longevity, male and female body size, fecundity, male fertility, flight ability and mating capacity | Bond et al. (2017) |
| 7.5 litre container (height of 24cm and diameter 23.5cm) | 5 litres of tap water | 500 | 0.1 larvae/ml | Temperature and Food | Mean days to pupation, proportion of mosquitoes which survived to pupation, hatch success of second gonotrophic cycle, Infection rates of DENV-1 from infected blood meal and if vertical transmission of DENV-1 occurred. | Buckner et al. (2016) |
| 30 x 21 x 5.5cm | 450ml | 200 | 0.44 larvae/ml | Correlating nutritional reserves with critical weight | Critical weight (the weight where 50% of the larvae pupated) and nutritional reserves of fourth instar larvae (measured by the amount of glycogen and sugar present) | Chambers and Klowden (1990) |
| 19 x 13 x 6cm | 450ml | 40 | 0.089 larvae/ml | Temperature | Survival rates between first and fourth instar larvae | Chang et al. (2007) |
| Dimensions unspecified. Only states a 473ml container | 250ml | 10, 20, 40 or 80 larvae | 0.04 larvae/ml,  0.08 larvae/ml,  0.16 larvae/ml, 0.32 larvae/ml | Density, temperature and diet | Time to pupation, time to emergence and mortality rates during development. | Couret et al. (2014) |
| Unspecified. Plastic tubes | 10ml | 1 | 0.1 larvae/ml | Temperature | Survival, sex and wing length (as a proxy for bodysize). | De Majo et al. (2021) |
| **Larval rearing tray size** | **Volume of water used per tray** | **Number of larvae per tray** | **Density (Larvae per millilitre)** | **Focus of experiment** | **Traits looked at** | **Reference** |
| Cylinders of 50mm diameter | 30ml | 21, ,16, 64 | 0.70 larvae/ml,  0.53 larvae/ml, 2.13 larvae/ml | Chemical interference | Time to reach each instar | Dye (1982) |
| Cylinders of 50mm diameter | 30ml | 64 | 2.13 larvae/ml | Chemical interference | Duration of first instar larvae period | Dye (1984) |
| Dimensions unspecified. Only states a 500ml tub. | 200ml | 20 | 0.1 larvae/ml | Temperature and diet | Mortality rates, development time (hatching to emergence) and male and female wing size | Farjana et al. (2012) |
| 27 x 19 x 7cm | 1 litre | 150, 500, 1200 | 0.15 larvae/ml,  0.5 larvae/ml, 1.2 larvae/ml | Density | Development time (time to pupation), dead larvae, dead pupae, male and female wing size, larvae to adult, pupae to adult survival  and larvae to pupae survival | Gama et al. (2005) |
| 25 x 25 x 7cm | 500ml | 750 | 1.5 larvae/ml | Diet | Larvae to pupae development time, pupae to adult development time, sex ratio, 4^th^ instar larvae body size, pupae body size, adult male wing size, adult female wing size, fecundity and egg hatch success | Gunathilaka et al. (2018) |
| 25 x 25 x 7cm | 500ml | 400 | 0.8 larvae/ml | Diet | Development time (time to pupation and time to emergence), sex, larvae morphometric parameters (head length, head width, thoracic length, thoracic width, abdominal length, abdominal width and total length), survival (percentage of larvae which emerged as pupae, percentage of adults emerged from pupae), duration of adult survival (longevity of adults), number of eggs laid, wing length and adult morphometric parameters (thorax width and length), biting frequency and vectorial capacity. | Gunathilaka et al. (2019) |
| **Larval rearing tray size** | **Volume of water used per tray** | **Number of larvae per tray** | **Density (Larvae per millilitre)** | **Focus of experiment** | **Traits looked at** | **Reference** |
| Unspecified | Unspecified | 200 and 100 | N/A | Effect of blood meal, limited larval diet and limited diet on lifespan | Adult lifespan | Joy et al. (2010) |
| Unspecified | 500ml | Approx 200 larvae | 0.4 larvae/ml | Diet | Mating success, male wing size, male adult  survival and swarming activity | Lang et al. (2018) |
| 500ml container | 400ml | 100 | 0.25 larvae/ml | Effects of temperature on *Wolbachia-*uninfected and *Wolbachia*-infected Ae. aegypti | Pupae and adult eclosion proportions, wing length (indication of body size), fecundity (number of eggs laid and proportion of eggs which hatched), Wolbachia density | Lau et al. (2020) |
| 250ml plot (8cm diameter and 6cm depth) | 175ml dechlorinated water | 4,8,16,32,64, 128,256 larvae per pot | 0.023 larvae/ml,  0.046 larvae/ml,  0.091 larvae/ml,  0.18 larvae/ml,  0.37 larvae/ml,  0.73 larvae/ml,  1.46 larvae/ml | Density | Development time (time from larvae to pupae), survival (larvae to adults), pupal weight and biomass of all pupae in each treatment | Maciá (2009) |
| Unspecified | 2L | 200 | 0.2 larvae/ml | Diet availability on bacterial community size and composition in larvae water, larvae and adults | Adult wing length (proxy for bodysize), total bacterial load and bacterial community composition in breeding water, whole larvae and adult mosquito midguts | MacLeod et al. (2021) |
| Dimensions unspecified. Only states a  250ml beaker | 150ml | 30-40 eggs | 0.23 larvae/ml (averaged at 35  eggs per  beaker) | Temperature | Egg hatch success | Mohammed and Chadee (2011) |
| Dimensions unspecified. Only states a 1L beaker | 800mL | 100 | 0.125 larvae/ml | Temperature | Rate of pupation, sex ratio, male wing size and female wing size. | Mohammed and Chadee (2011) |
| **Larval rearing tray size** | **Volume of water used per tray** | **Number of larvae per tray** | **Density (Larvae per millilitre)** | **Focus of experiment** | **Traits looked at** | **Reference** |
| 38 x 23 x 6cm | 1.5 litres | 200 or 1200 | 0.13 larvae/ml,  0.8 larvae/ml | Density | Time to pupate (for males and females), immature stage mortality rates and fecundity | Moore and Fisher (1969) |
| Unspecified for rearing for L1 to late L3 or L4.  Late L3 or L4 – beaker, dimensions unspecified. | Unspecified for rearing for L1 to late L3 or L4.  Late L3 or L4 250ml | Unspecified for rearing for L1 to late L3 or L4.  Late L3 or L4 20 | L1 to late L3/L4 N/A  Late L3 or L4 0.08 larvae/ml | Temperature on *Ae. Aegypti* response to pyriproxyfen | Inhibition of adult emergence (recorded as larvae and pupae mortality) and larval development time. | Moura et al. (2021) |
| Glass bowl (11.5cm  diameter, 5cm deep) | 200ml | 50 | 0.25 larvae/ml | Diet and relationship between wing length and dry weight | Wing length, dry weight | Nasci (1990) |
| 400ml cups | 350ml | 20, 40 | 0.057 larvae/ml,  0.11 larvae/ml | Intra-specific competition and density | Larval to adult survival for males and females, female wing length, median female time to eclosion, development time (larvae to adult), female feeding success (including the mean number of bloodmeals, mean life time fecundity, mean length of first egg production cycle, mean fecundity  and mean blood-feeding frequency). | Noden et al. (2016) |
| 35.6 x 27.9 x 8.3cm | 1L | 200 | 0.2 larvae/ml | Temperature | Number of eggs laid, hatch rates of eggs produced, wing length, larvae development, larvae, pupae and adult survival and midgut microbes. | Onyango et al. (2020) |
| Dimensions unspecified. Only states a 20 litre bucket | At least 19.5 litres of water | 20 | 0.0001 larvae/ml | Food and Temperature interaction | Pupae development time (days to pupation) and wing area | Padmanabha et al. (2011) |
| **Larval rearing tray size** | **Volume of water used per tray** | **Number of larvae per tray** | **Density (Larvae per millilitre)** | **Focus of experiment** | **Traits looked at** | **Reference** |
| 27 x 20 x 7.5cm | 1 litre | 75 and 750 | 0.075 larvae/ml,  0.75 larvae/ml | Effects of male age and body size on sperm  number | The total number of spermatozoa in testes and seminal vesicles | Ponlawat and Harrington (2007) |
| 18 x 30 x 4.5 cm | 750ml | 25 (low), 50  (medium), 100 (high) larvae | 0.033 larvae/ml,  0.067 larvae/ml,  0.13 larvae/ml, | Density | Adult female longevity | Reiskind and Lounibos (2009) |
| 1000ml cylinders, 105mm diameter and  125.5mm height | 800ml | 30 | 0.038 larvae/ml | Diet | Time to pupation, time to adult emergence, sex ratio, wing length and survival (for each instar and pupae to adult survival) | Romeo Aznar et al. (2018) |
| Unspecified | 7ml | 1 | 0.14 larvae/ml | Temperature | Larval length, larval head width, larval wet weight, wet weight of pupae, wet weight of adults for each sex, survival for each instar  and stage and development time | Rueda et al. (1990) |
| 17 x 12 x 5cm | 200ml | 200, 400, 800,  1600 | 1 larvae/ml, 2 larvae/ml, 4 larvae/ml, 8 larvae/ml | Food amount, Density and  temperature | Larvae to pupae survival, time for 50% of larvae to pupate and time for 90% of larvae to pupate | Russell (1986) |
| Unspecified | 1L | 200 | 0.2 larvae/ml | Temperature exposure impact on the susceptibility to insecticides as adult | Female mortality | Salinas et al. (2021) |
| 24 well microtiter plate (1.5cm diameter and 1.5cm depth) | Unspecified | 1 per well | N/A | Diet type x temperature | Development time (larvae to pupae and pupae to adult), survival (percentage pupation and adult eclosion), sex, pupal morphometrics (length of cephalothorax), adult morphometrics (wing length), male longevity, teneral energy reserves (measured sugar content), mating capacity, heat and cold tolerance (upper and lower lethal temperatures that would cause 50% mortality in adult male population) | Sasmita et al. (2019) |
| **Larval rearing tray size** | **Volume of water used per tray** | **Number of larvae per tray** | **Density (Larvae per millilitre)** | **Focus of experiment** | **Traits looked at** | **Reference** |
| 25 x 25 x 7cm | 500ml | 250 | 0.5 larvae/ml | Diet type | Morphometric parameters of: L4 larvae (head length, head width, thoracic length, thoracic width, abdominal length, abdominal width, total length), pupae (cephalothoracic length and width) and adult (thoracic length, thoracic width, abdominal length and abdominal width), survival (percentage of larvae which pupated and percentage of pupae which emerged as adults) adult wing length, number of days for 50% of male adults in each treatment to die (male longevity), flight ability, number of eggs laid and hatch rate of eggs. | Senevirathan et al. (2020) |
| Unspecified | 250ml | 10, 80, 200 | 0.04, 0.32 and 0.8 larva/ml | Larval rearing conditions on lipid metabolism for synthesis and mobilization of TAG stored | Reproduction (number of eggs laid), fasting (amount of TAG (triacylglycerol reserves) determined), protein concentration, lipid mobilization, body size (wing length used as a proxy) | Silva et al. (2021) |
| 22.5 x 12.8 x 3.59cm | 250ml | 150 | 0.6 larvae/ml | Diet (nutrition) | Development rate (time to pupation and adult emergence), survival (L1 to pupae, pupae to adults and L1 to adults), sex ratio, wing length (proxy for body size), ingestion rates and nutrition reserve accumulation | Souza et al. (2019) |
| 25mm diameter x 95mm tall | 20ml | 4, 5, 6, 7 or 8 | 0.2 larvae/ml,  0.25 larvae/ml,  0.3 larvae/ml,  0.35 larvae/ml,  0.4 larvae/ml | Food and density | Percentage survival, mass and age of prime male and prime female at pupation and average mass of males and females at pupation. The prime individual is defined as one male and one female per treatment which was expected to have the greatest  reproductive success. | Steinwascher (2018) |
| **Larval rearing tray size** | **Volume of water used per tray** | **Number of larvae per tray** | **Density (Larvae per millilitre)** | **Focus of experiment** | **Traits looked at** | **Reference** |
| 9cm high and 9cm diameter cylinders | 5cm height (approx 300ml) | 30 | Approx 0.1 larvae/ml | Shading (light intensity) and temperature | Hatch rate, development time and survival. | Sukiato et al. (2019) |
| 17 x 12 x 6cm | 500ml | 50 larvae | 0.1 larvae/ml | Temperature | Larval development, male wing size and  female wing size | Tun-Lin et al. (2000) |
| 34.3 x 25.4 x 3.8cm | 600ml | 200 | 0.33 larvae/ml | Diet composition | Survival (proportion of larvae reaching pupation), development time (time from larvae to pupation), adult body weight, adult wing length and adult nutritional stores (sugar, glycogen and lipids) | Van Schoor et al. (2020) |
| Unspecified | 100ml | Ranged from 1 to 128 per cup | Range from  0.01 larvae/ml to  1.28 larvae/ml | Diet and density | Larvae mortality, pupae mortality and sex of pupae | Wada (1965) |
| Unspecified | 97.5, 17.5,7.5,  2.5 and 1.5ml | 20 | 0.2, 1, 2, 4, and  5 larvae per ml | Temperature and density | Larval mortality, pupae mortality, time for first larvae to pupate, time for the first adult to emerge and the total number of adults  which emerged | Zapletal et al. (2018) |
| Unspecified | 3ml | 1 | 0.33 larvae/ml | Food variability | Larval growth rates (measured using body size), adult wing length, time until adult emergence, adult longevity, adult survival  and fecundity | Zeller and Koella (2016) |

References:

1. Achari TS, Panda C, Barik TK. Biochemical response of *Aedes aegypti* and *Aedes albopictus* mosquitoes after exposure to thermal stress and toxin of Bacillus thuringiensis israelensis. Int J Trop Insect Sci. 2022;42: 651-660.
2. Agnew P, Hide M, Sidobre C, Michalakis Y. A minimalist approach to the effects of density-dependent competition on insect life-history traits. Ecol Entomol. 2002;27: 396-402.
3. Alto BW, Reiskind MH, Lounibos LP. Size Alters Susceptibility of Vectors to Dengue Virus Infection and Dissemination. AM J Trop Med Hyg. 2008;79: 688-695.
4. Arrivillaga J, Barrera R. Food as a limiting factor for *Aedes aegypti* in water-storage containers. J Vector Ecol. 2004;29: 11-20.
5. Bargielowski I, Nimmo D, Alphey L, Koella JC. Comparison of Life History Characteristics of the Genetically Modified OC513A Line and a Wild Type Strain of *Aedes aegypti*. Plos One. 2011;6: e20699.
6. Bedhomme S, Agnew P, Sidobre C, Michalakis Y. Virulence reaction norms across a food gradient. P Roy Sox B-Biol Sci. 2004;271: 739-744.
7. Bond JG, Ramirez-Osorio A, Marina CF, Fernandez-Salas I, Liedo P, Dor A, et al. Efficiency of two larval diets for mass-rearing of the mosquito *Aedes aegypti*. Plos One. 2017;12: e0187420
8. Buckner EA, Alto BW, Lounibos LP. Larval Temperature-Food Effects on Adult Mosquito Infection and Vertical Transmission of Dengue-1 Virus. J Med Entomol. 2016;53: 91-98.
9. Chambers G, Klowden M. Correlation of Nutritional Reserves with a Critical Weight for Pupation in Larval *Aedes aegypti* Mosquitos. J Am Mosq Control Assoc. 1990;6: 394-399.
10. Chang LE, Hsu T, Teng H, Ho C. Differential survival of *Aedes aegypti* and *Aedes albopictus* (Diptera : Culicidae) larvae exposed to low temperatures in Taiwan. J Med Entomol. 2007;44: 205-210.
11. Couret J, Dotson E, Benedict MQ. Temperature, larval diet, and density effects on development rate and survival of *Aedes aegypti* (Diptera: Culicidae). PLoS One. 2014;9: e87468.
12. De Majo MS, Zanotti G, Gimenez JO, Campos RE, Fischer S. Comparative Study on the Thermal Performance of Three *Aedes aegypti* (Diptera: Culicidae) Populations From Argentina. J Med Entomol. 2021;58: 1733-1739.
13. Dye C. Intraspecific Competition Amongst Larval *Aedes aegypti* – Food Exploitation Or Chemical Interference. Ecol Entomol. 1982;7: 39–46.
14. Dye C. Competition Amongst Larval *Aedes aegypti* – the Role of Interference. Ecol Entomol. 1984;9: 355-357.
15. Farjana T, Tuno N, Higa Y. Effects of temperature and diet on development and interspecies competition in *Aedes aegypti* and *Aedes albopictus*. Med Vet Entomol. 2012;26: 210-217.
16. Gama R, Alves K, Martins R, Eiras A, de Resende M. Effect of larvae density on adult size of *Aedes aegypti* reared under laboratory conditions. Rev Soc Bras Med Trop. 2005;38: 64-66.
17. Gunathilaka N, Upulika H, Udayanga L, Amarasinghe D. Effect of Larval Nutritional Regimes on Morphometry and Vectorial Capacity of *Aedes aegypti* for Dengue Transmission. Biomed Res Int. 2019;2019: 3607342.
18. Gunathilaka PADHN, Uduwawala UMHU, Udayanga NWBAL, Ranathunge RMTB, Amarasinghe LD, Abeyewickreme W. Determination of the efficiency of diets for larval development in mass rearing *Aedes aegypti* (Diptera: Culicidae). Bull Entomol Res. 2018;108: 583-593.
19. Joy TK, Arik AJ, Corby-Harris V, Johnson AA, Riehle MA. The impact of larval and adult dietary restriction on lifespan, reproduction and growth in the mosquito *Aedes aegypti*. Exp Gerontol. 2010;45: 685-690.
20. Lang B, Igdobe S, McMullen K, Drury F, Qureshi A, Cator L. The effect of larval diet on adult survival, swarming activity and copulation success in male *Aedes aegypti* (Diptera: Culicidae). J Med Ent. 2018;55: 29-35.
21. Lau MJ, Ross PA, Endersby-Harshman NM, Hoffmann AA. Impacts of Low Temperatures on Wolbachia (Rickettsiales: Rickettsiaceae)-Infected *Aedes aegypti* (Diptera: Culicidae). J Med Entomol. 2020;57: 1567-1574.
22. Maciá A. Effects of larval crowding on development time, survival and weight at metamorphosis in *Aedes aegypti* (Diptera: Culicidae). Rev Soc Entomol Argent. 2009;68: 107-114.
23. MacLeod HJ, Dimopoulos G, Short SM. Larval Diet Abundance Influences Size and Composition of the Midgut Microbiota of *Aedes aegypti* Mosquitoes. Front Microbiol. 2021;12: 645362.
24. Mohammed A, Chadee DD. Effects of different temperature regimens on the development of *Aedes aegypti* (L.) (Diptera: Culicidae) mosquitoes. Acta Trop. 2011;119: 38-43.
25. Moore CG, Fisher BR. Competition in Mosquitoes. Density and Species Ratio Effects on Growth, Mortality, Fecundity, and Production of Growth Retardant. Ann Entomol Soc Am. 1969;62: 1325-1331.
26. Moura L, de Nadai BL, Bernegossi AC, Felipe MC, Castro GB, Corbi JJ. Be quick or be dead: high temperatures reduce *Aedes aegypti* (Diptera: Culicidae) larval development time and pyriproxyfen larvicide efficiency in laboratory conditions. Int J Trop Insect Sci. 2021;41: 1667-1672.
27. Nasci R. Relationship of Wing Length to Adult Dry-Weight in several Mosquito Species (Diptera, Culicidae). J Med Entomol. 1990;27: 716:719.
28. Noden BH, O’Neal PA, Fader JE, Juliano SA. Impact of inter- and intra- specific competition among larvae on larval, adult and life-table traits of *Aedes aegypti* and *Aedes albopictus* females. Ecol Entomol. 2016;41: 192-200.
29. Onyango GM, Bialosuknia MS, Payne FA, Mathias N, Ciota TA, Kramer DL. Increase in temperature enriches heat tolerant taxa in *Aedes aegypti* midguts. Sci Rep. 2020;10: 19135.
30. Padmanabha H, Bolker B, Lord CC, Rubio C, Lounibos LP. Food Availability Alters the Effects of Larval Temperature on *Aedes aegypti* Growth. J Med Entomol. 2011;48: 974-984.
31. Ponlawat A, Harrington LC. Age and body size influence male sperm capacity of the dengue vector *Aedes aegypti* (Diptera: Culicidae). J Med Entomol. 2007;44: 422-426.
32. Reiskind MH, Lounibos LP. Effects of intraspecific larval competition on adult longevity in the mosquitoes *Aedes aegypti* and *Aedes albopictus*. Med Vet Entomol. 2009;23: 62-68.
33. Romeo Aznar A, Alem I, Sol De Majo M, Byttebier B, Solari HG, Fischer S. Effects of scarcity and excess of larval food on life history traits of *Aedes aegypti* (Diptera: Culicidae). J Vector Ecol. 2018;43: 117-124.
34. Rueda L, Patel K, Axtell R, Stinner R. Temperature-Dependent Development and Survival Rates of *Culex quinquefasciatus* and *Aedes aegypti* (Diptera, Culicidae). J Med Entomol. 1990;27: 892-898.
35. Russell R. Larval Competition between the Introduced Vector of Dengue Fever in Australia, *Aedes aegypti* (L), and a Native Container-Breeding Mosquito, *Aedes notoscriptus* (Skuse) (Diptera, Culicidae). Aust J Zool. 1986; 34: 527-534.
36. Salinas WS, Feria-Arroyo TP, Vitek CJ. Temperatures Influence Susceptibility to Insecticides in *Aedes aegypti* and *Aedes albopictus* (Diptera: Culicidae) Mosquitoes. J Pathog. 2021;10: 992.
37. Sasmita HI, Tu WC, Bong LJ, Neohh KB. Effects of larval diets and temperature regimes on life history traits, energy reserves and temperature tolerance of male *Aedes aegypti* (Diptera: Culicidae): optimizing rearing techniques for the sterile insect programmes. Parasites Vectors. 2019;12: 578.
38. Senevirathna U, Udayanga L, Ganehiarachchi G, Hapugoda M, Ranathunge T, Gunawardene NS. Development of an Alternative Low-Cost Larval Diet for Mass Rearing of *Aedes aegypti* Mosquitoes. BioMed Res Int. 2020;2020: 1053818.
39. Silva E, Santos LV, Caiado MS, Hastenreiter LSN, Fonseca SRR, Carbajal-de-la-Fuente AL, et al. The influence of larval density of triacylglycerol content in *Aedes aegypti* (Linnaeus) (Diptera: Culicidae). Arch Insect Biochem Physiol. 2021;106: e21757.
40. Souza RS, Virginio F, Riback TIS, Suesdek L, Barufi JB, Genta FA. Microorganism-Based Larval Diets Affect Mosquito Development, Size and Nutritional Reserves in the Yellow Fever Mosquito Aedes aegypti (DipteraL Culicidae). Front Physiol. 2019;10: 152.
41. Steinwascher K. Competition among *Aedes aegypti l*arvae. PLoS one. 2018;13: e0202455.
42. Sukiato F, Wasserman RJ, Foo SC, Wilson RF, Cuthbert RN. The effects of temperature and shading on mortality and development rates of *Aedes aegypti* (Diptera: Culicidae). 2019;44: 264-270.
43. Tun-Lin W, Burkot T, Kay B. Effects of temperature and larval diet on development rates and survival of the dengue vector Aedes aegypti in north Queensland, Australia. Med Vet Entomol. 2000;14: 31-37.
44. van Schoor T, Kelly ET, Tam N, Attargo GM. Impacts of Dietary Nutritional Composition on Larval Development and Adult Body Composition in the Yellow Fever Mosquito (*Aedes aegypti*). Insects. 2020;11: 535.
45. Wada Y. Effect of Larval Density on the Development of *Aedes aegypti* (L.) and the Size of Adults. Quaest Entomol. 1965;1: 223-249.
46. Zapletal J, Erraguntla M, Adelman NZ, Myles KM, Lawley MA. Impacts of diurnal temperatures and larval density on aquatic development of *Aedes aegypti*. Plos One. 2018; 13: e0194025.
47. Zeller M, Koella JC. Effects of food variability on growth and reproduction of *Aedes aegypti*. Ecol Evol. 2016;6: 552-559.
