# Supplementary material for: The size of larval rearing container modulates the effects of diet amount and larval density on larval development in Aedes aegypti
Source: PLoS One. 2023 Jan 25;18(1):e0280736. doi: 10.1371/journal.pone.0280736 (PMC9876358; doi:10.1371/journal.pone.0280736)
Supplement: S2 Table — (DOCX) [file pone.0280736.s002.docx]

|  |  | PI as measured | PI Development Constant | PI Fecundity Constant | PI Survival Constant | PI Survival and Fecundity Constant |
| --- | --- | --- | --- | --- | --- | --- |
| Container Size | df | 2, 29.86 | 2,33 | 2, 30.08 | 2, 29.78 | 2, 30.03 |
|  | F | 9.74 | 0.53 | 15.13 | 9.75 | 13.14 |
|  | P | **<0.001** | 0.60 | **<0.001** | **<0.001** | **<0.001** |
|  | η^2^ | **0.07** | 0.27 | **0.10** | **0.08** | **0.10** |
| Diet | df | 2, 29.19 | 2,33 | 2, 29.25 | 2, 29.18 | 2, 29.25 |
|  | F | 131.52 | 9.20 | 144.02 | 116.08 | 111.25 |
|  | P | **<0.001** | **<0.001** | **<0.001** | **<0.001** | **<0.001** |
|  | η^2^ | **0.91** | **0.46** | **0.87** | **0.91** | **0.87** |
| Container Size x Diet | df | 4, 29.19 | 4, 33 | 4, 29.25 | 4, 29.18 | 4, 29.25 |
|  | F | 1.45 | 1.85 | 1.64 | 1.29 | 1.47 |
|  | P | 0.24 | 0.14 | 0.19 | 0.30 | 0.24 |
|  | η^2^ | 0.02 | 0.28 | 0.02 | 0.02 | 0.02 |
|  |  |  |  |  |  |  |
| Container Size | df | 2, 16.42 | 2,19 | 2, 19 | 2, 16.43 | 2, 16.68 |
|  | F | 1.53 | 0.74 | 1.67 | 1.63 | 1.11 |
|  | P | 0.24 | 0.49 | 0.21 | 0.23 | 0.35 |
|  | η^2^ | 0.06 | 0.27 | 0.07 | 0.05 | 0.04 |
| Density | df | 1, 15.62 | 1,19 | 1, 19 | 1, 15.67 | 1, 15.95 |
|  | F | 36.67 | 2.51 | 27.14 | 45.64 | 31.10 |
|  | P | **<0.001** | 0.13 | **<0.001** | **<0.001** | **<0.001** |
|  | η^2^ | **0.65** | 0.45 | **0.57** | **0.68** | **0.62** |
| Container Size x Density | df | 2, 15.61 | 2,19 | 2, 19 | 2, 15.65 | 2, 15.93 |
|  | F | 8.26 | 0.76 | 8.54 | 8.93 | 2.47 |
|  | P | **0.003** | 0.48 | **0.002** | **<0.001** | **0.003** |
|  | η^2^ | **0.29** | 0.28 | **0.36** | **0.27** | **0.34** |
