# Supplementary material for: The size of larval rearing container modulates the effects of diet amount and larval density on larval development in Aedes aegypti
Source: PLoS One. 2023 Jan 25;18(1):e0280736. doi: 10.1371/journal.pone.0280736 (PMC9876358; doi:10.1371/journal.pone.0280736)
Supplement: S1 Fig — (DOCX) [file pone.0280736.s003.docx]

**
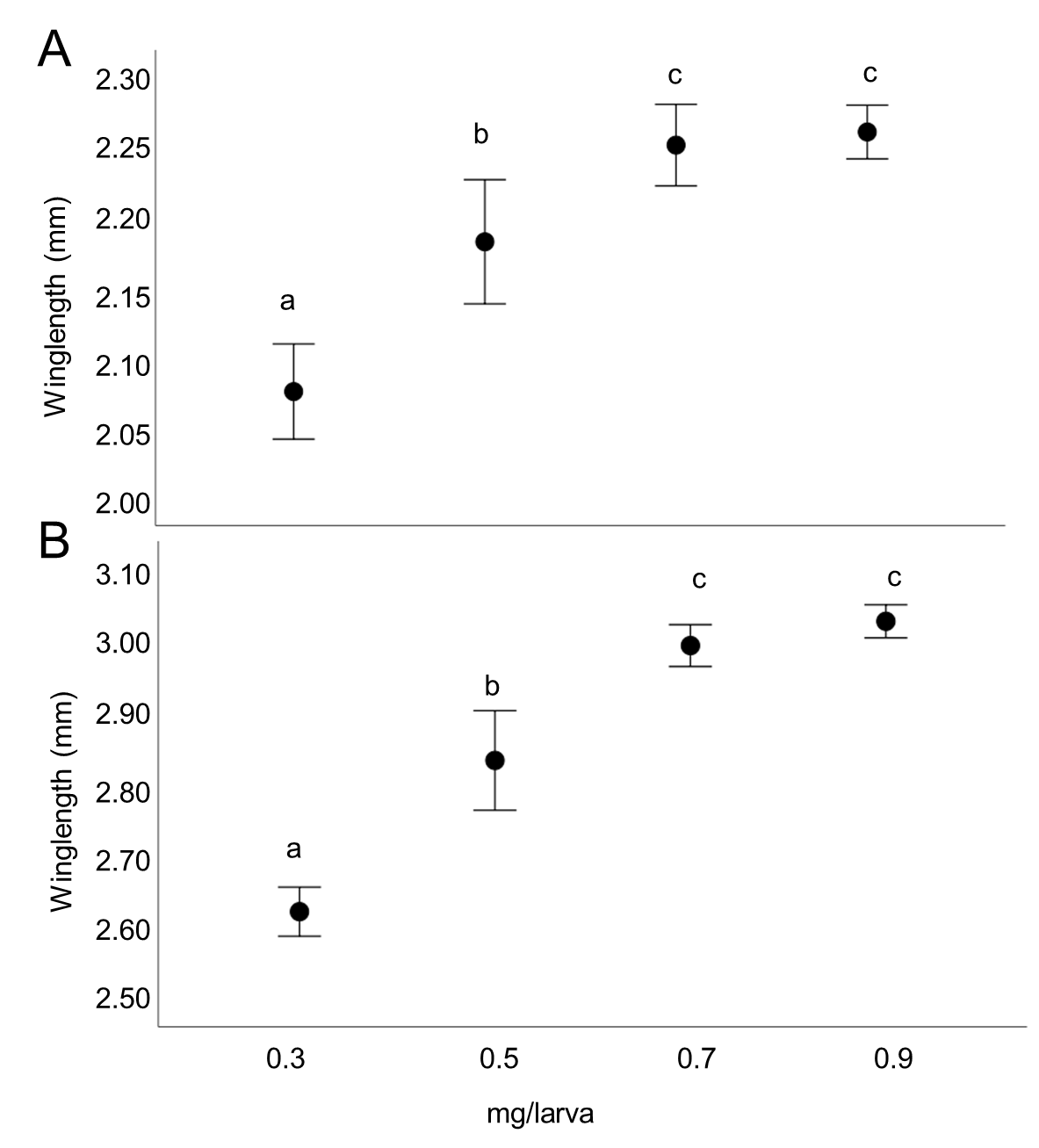
**

**Figure S1.** Pilot data on effect of food mg/ml on male and female winglength. Newly hatch 1^st^ instar larvae were transferred into 0.5 ml of water held in 18.0 x 11.0 x 6.0 cm trays. Groups of 100 larvae were provided 0.3, 0.5, 0.7, or 0.9 mg of diet per larva per day. Larvae in each tray were counted daily to determine the appropriate food amount. A. Winglengths of males emerging from each treatment B. Winglengths of females emerging from each treatment. n=2 replicate trays per treatment with >30 individuals per treatment. Letters indicate significance in pairwise comparison with Bonferroni correction. Error bars represent + 95% Confidence Intervals
